# Phages and quorum sensing: findings to consider in phage therapy

**DOI:** 10.1007/s10096-025-05375-3

**Published:** 2025-12-03

**Authors:** Laura Fernández-Garcia, Lucia Blasco, Inés Bleriot, Lucía Arman, Clara Ibarguren-Quiles, Antonio Barrio-Pujante, Manuel González de Aledo, Rodolfo García-Contreras, Rafael Cantón, Thomas K. Wood, María Tomas

**Affiliations:** 1 Microbiology Translational and Multidisciplinary (MicroTM)-Research Institute Biomedical A Coruña (INIBIC) and Microbiology Department of Hospital A Coruña (CHUAC), University of A Coruña (UDC), A Coruña, Spain; 2 Servicio de Microbiología, Hospital Universitario Ramón y Cajal and Instituto Ramón y Cajal de Investigación Sanitaria (IRYCIS), Madrid, Spain; 3 Microbiology and Parasitology Department Faculty of Medicine, UNAM, Mexico City, Mexico; 4 Department of Chemical Engineering, Pennsylvania State University, University Park, PA USA; 5 Study Group on Mechanisms of Action and Resistance to Antimicrobials (GEMARA) on behalf of the Spanish Society of Infectious Diseases and Clinical Microbiology (SEIMC), Málaga, Spain; 6 CIBER de Enfermedades Infecciosas (CIBERINFEC), Instituto de Salud Carlos III, Madrid, Spain; 7 MePRAM, Proyecto de Medicina de Precisión contra las resistencias Antimicrobianas, Madrid, Spain

**Keywords:** Quorum sensing, Phage infection, Bacterial defence, Phage therapy

## Abstract

**Purpose:**

This review aims to provide an overview of current knowledge on the involvement of QS in phage infection. The role of QS in bacterial defence against phages is emphasized, without overlooking the fact that QS can sometimes also promote phage infection. We also review the implications of QS in phage therapy and current perspectives.

**Methods:**

For the bibliographic review, PubMed and Google Scholar were used to search for publications on “quorum-sensing” and “phage infection”.

**Results:**

The relationships between bacteria and phages are extremely complicated and involve several mechanisms. Quorum sensing (QS) is a communication system involved in controlling bacterial fitness, both at population and individual levels. Phages (viruses that infect bacteria) play a major role in the natural regulation of bacterial populations. In order to protect themselves, bacteria have developed several defence mechanisms involving different levels of protection, such as prevention of phage entry and phage assembly, degradation of phage nucleic acids, and entrance in a dormant state (persistence). QS has recently been shown to affect some of these phage defence mechanisms. In this review, the main influence of QS in phage infection is discussed. Finally, some innovative treatment approaches, including using engineered phages harbouring T7aiiA QQ enzyme and QS inhibitors such as SsoPox-W2631 and penicillinic acid, are also considered. However, it is important to note that the use of QS-interfering molecules may also reduce the efficacy of phage therapy.

**Conclusion:**

QS is an important mechanism that affects several bacterial metabolisms, particularly in phage defence. Despite the complex interaction between QS and phages, modifying QS has been found to enhance phage therapy.

## Introduction

Phages, viruses that infect bacteria, are considered a major natural regulator of bacterial populations. In order to manage this, they have two types of life cycles: the lysogenic cycle, in which the phage genome becomes integrated into the bacterial genome; and the lytic cycle, in which the phage enters the host, uses the bacterial machinery to replicate itself and then lyses the host to release its progeny [1]. Due to the constant interaction between phages and their bacterial hosts, both are permanently updating their defence/counter-defence strategies [2].

The use of phage therapy has recently been proposed as a promising alternative to antibiotic therapy. The increase in multiresistant bacteria for which no viable antibiotic treatments are available has become increasingly common in the last few decades and is now a worldwide health problem [3]. Clinical trials are being developed and legal regulations implemented to enable the use of phage therapy in hospitalized patients in several countries [4]. One of the requirements of phage therapy is the use of strictly lytic phages, as the nucleic acid of this type of phage will not become integrated in the bacterial genome, thus preventing horizontal gene transfer of virulence and resistant genes [5, 6]. Additionally, phage therapy is considered a safe and very specific treatment. Phages have a narrow spectrum of action, being able to infect only those bacterial targets that carry specific receptors [7]. This specificity enables directed treatment against the pathogen, evading commensal bacteria [4, 6].

Phage multiplication is directly related to the number of bacteria available. Thus, the greater the number of bacteria, the greater the number of potential hosts and the greater the number of new phages produced, which in turn increases phage pressure, driving bacterial resistance [8, 9]. Phage progeny rates have been shown to increase in logarithmic phase. Phages spread readily under conditions of high cell density (HCD) [10], although the bacterial quorum sensing (QS) communication system is also activated under such conditions [11]. Nevertheless, it is worth mentioning that HCD conditions might contribute to bacterial starvation conditions, significantly reducing bacterial activity and therefore, substantially reducing phage susceptibility [12, 13]. Bacteria have developed several phage-resistant mechanisms to protect themselves, especially during slow infection cycles, during which the bacteria in the population promote the activation of QS and related defence mechanisms. These strategies can be classified according to their mechanism of action into the following: prevention of phage adsorption, prevention of phage assembly, degradation of phage nucleic acid, and increase in the number of persister cells [14]. However, phages have developed anti-defence mechanisms that allow them to overcome the mentioned bacterial defences, keeping both bacteria and phages in a constant arms race [15].

The QS communication system is based on secreted signals (auto-inducers, AIs) that enable bacteria to determine population density and activate metabolic routes that are too energetically expensive to be activated individually (i.e., biofilm) [16]. QS is based on the accumulation of molecules, so that when there are few bacteria in the population, the number of AIs in the environment will not be enough to activate QS. However, as the population increases, accumulation of AIs in the environment can activate QS and related mechanisms [17]. The QS system is a global regulator that has been related to several bacterial mechanisms, ranging from bioluminescence to resistance and including motility, biofilm formation, and virulence [18]. The regulation is context-dependent, i.e., during the first stage of biofilm formation (attachment), QS promotes motility and also production of fimbriae; during the following steps (microcolony formation and maturation), the QS system represses these mechanisms and promotes polysaccharide excretion to form the biofilm matrix [19]. All QS systems have four main characteristics: (i) production of diffusible molecules, AIs; (ii) production of cytoplasmatic or inter-membrane receptors specific for the AIs; (iii) auto-stimulated production of AIs; and (iv) regulation of several bacterial mechanisms [20]. In addition to positive autoregulation, QS can also undergo negative autoregulation in order to balance the cost/benefit of resources, reducing the expression of QS-related genes when the bacterial population is not large enough to develop cooperative responses [21]. For this type of communication, bacteria use several types of AIs, including autoinducing peptides (AIPs), acyl-homoserine lactones (AHL) (previously known as AI-1), CAI-1 (*S*−3-hydroxytridecan-4-one), quinolones, autoinducer-2 (AI-2), and leaderless communication peptides [20, 22, 23]. The signals vary depending on the bacterial species, although many bacteria can detect signals from other species, such as *Escherichia coli*, which detects -but does not produce- AHL [24]. Some molecules related to cell communication, such as indole, are inter-kingdom, as well as inter-species, being detected and produced by bacteria, plants, and animals [23].

**Fig. 1:**
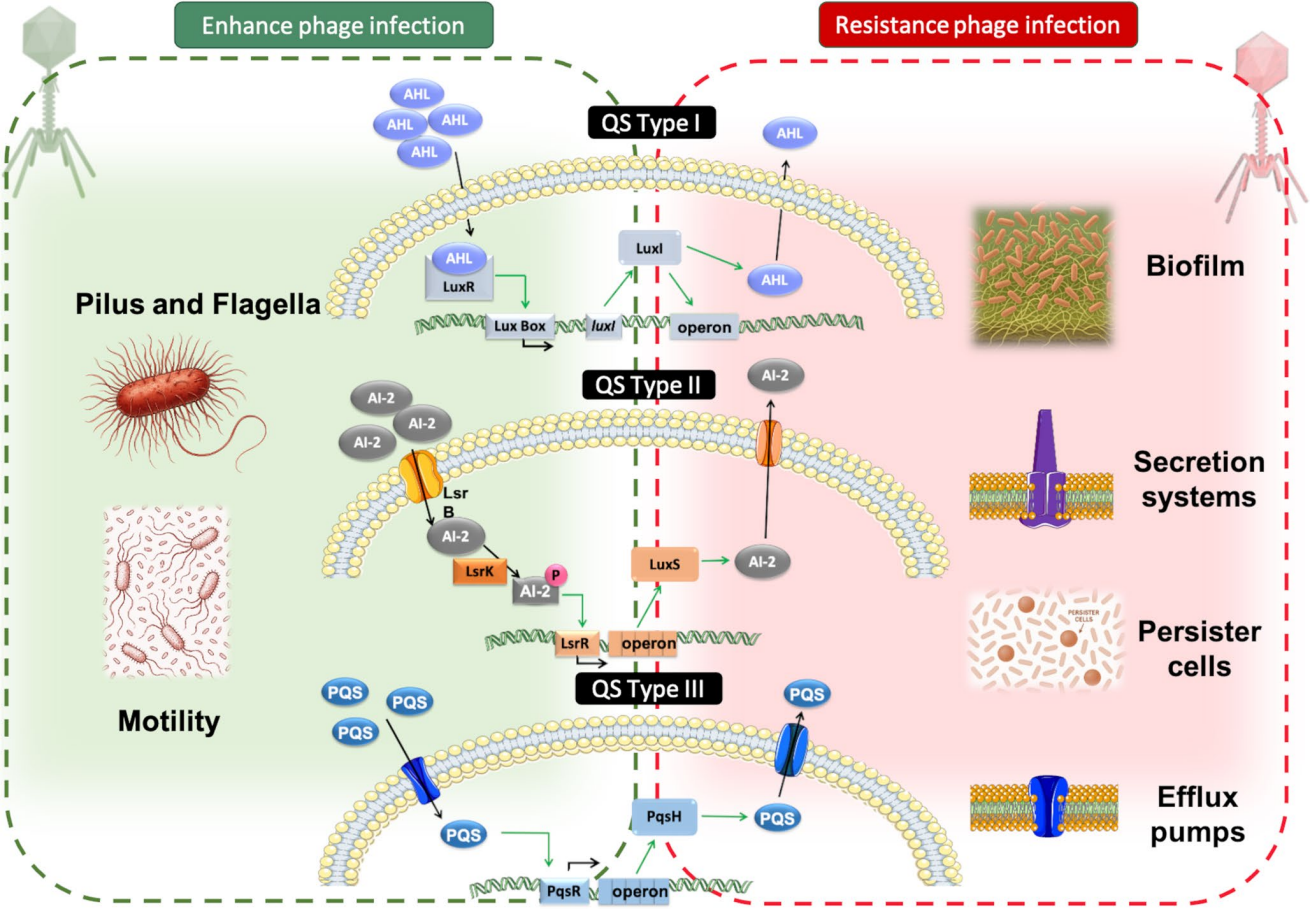
Graphical representation of the three main QS mechanisms and how the bacterial metabolism is affected. The green background highlights the mechanisms by which QS, depending on the situation, enhances phage infection, while the red background highlights those involved in phage resistance

The AI-2 based system is found in both Gram-positive and Gram-negative bacteria. Thus, AI-2 molecules are recognized by their membrane detectors (LuxP, LsrB, Cah-R, YeaJ) [25–27], entering the cytoplasm through transport channels and phosphorylating LsrK kinase, activating the Lsr operon, producing more AI-2, and regulating bacterial mechanisms [18]. Gram-negative bacteria have another two systems - one based on homoserine lactones (HSLs) and the other based on quinolones. In *Pseudomonas aeruginosa*, HSLs diffuse through the membrane and activate a LuxR-like transcriptional regulator (LasR or RhlR), which activates the Lux-like operon, including LuxI-like synthase (LasI or RhlI), to produce more signals. Conversely, the *Pseudomonas* quinolone signal (PQS) enters the cell through transporters to bind to the PqsR regulator, activating the Pqs operon [28, 29]. In Gram-positive bacteria, such as *Staphylococcus aureus*, the AIPs are recognized by a membrane histidine kinase, AgrC, which activates the accessory gene regulator (agr) cluster, leading to overexpression of exoproteins and downregulation of cell-wall-associated proteins [28]. Activating QS operons generates a cascade of activation affecting multiple important systems, which regulate several bacterial mechanisms involved in defence (biofilm and persister cell formation, efflux pump and secretion system activation), metabolism (bioluminescence, polysaccharide synthesis), and virulence (motility, toxin production), among others (Fig. 1) [30, 31]. In the last few decades, QS has been shown to regulate bacterial phage-defence mechanisms, including the following: (i) expression of surface proteins, (ii) biofilm formation, both of which are involved in adsorption inhibition; (iii) Clustered Regularly Interspaced Short Palindromic Repeats and associated proteins (CRISPR-CAS), involved in degrading phage nucleic acid; and (iv) toxin-antitoxin systems, involved in persister cell formation [17].

This mini-review aims to discuss the effects of QS in phage infection and how phages can control the host QS to their own benefit. In addition, some very promising molecules for use in phage therapy (based on recent findings) are mentioned. We consider here the complex interaction between QS and phages, and the possibility of exploiting this interaction to enhance phage therapy.

## Basic knowledge

Phages use specific receptors to infect bacteria. These receptors are surface structural proteins that are involved in several bacterial properties or mechanisms, including membrane structure, molecular transportation, and bacterial motility [32]. As already mentioned, QS is a global regulator of cell metabolism, affecting several mechanisms. The global regulation of bacterial metabolism affects, among other things, the genes involved in the synthesis of some of these structural proteins [33]. It is precisely through this regulation that the activation of QS in bacteria can be indirectly involved in phage infection. During the early stages of HCD, QS promotes bacterial motility (upregulating flagellar and pili genes, used for many phages as receptors). Under HCD (High Cell Density) conditions, QS can cause an increase in the secretion of bacterial extracellular matrix, reducing motility to promote the formation of biofilms, which thus hide phage receptors and prevent access to the host [34].

### QS as phage defence regulator

During phage infection, several stress responses are triggered in bacteria. One of the main molecules that bacteria activate under stress conditions is the alarmone signal or (p)ppGpp [35]. Stringent response (ppGpp) is known to activate QS [36]. It is possible that QS may be activated in response to phage infection through the stress response and (p)ppGpp.

One of the first defence mechanisms involves the modification or elimination of the membrane receptors, directly preventing phage adsorption. The reduction in receptors is closely related to QS. Although *E. coli* cannot produce AHLs, it can detect them through the SdiA regulator, which acts as a LuxR-type transcriptional regulator, regulating cell metabolism in response to external signals [24, 37, 38]. Through SdiA, *E. coli* strains have been observed to reduce susceptibility to phages such as λ and χ when exogenous AHLs are present in the environment. It has been demonstrated that in the presence of AHLs, *E. coli* reduced the amount of LamB protein, a maltose outer membrane channel used by phages as a receptor, and its depletion therefore reduces phage adsorption [39]. A similar increase in phage susceptibility was later observed when *sdiA* was deleted from *Klebsiella pneumoniae* strains. Although the authors did not observe any increase in phage resistance when C6-homoserine lactone (C6-HSL) was added to the cultures, proving that SdiA has a role independent from that of C6-HSL [40]. Moreover, *Vibrio cholerae* showed a reduction in the O1 antigen (highly variable surface polysaccharide region of Gram-negative bacteria [41]), used by many *Vibrio* phages as a receptor, when the high concentration of the auto-inducer is consistent with HCD conditions [42]. Likewise, other *Vibrio* spp. have shown similar responses to QS. Under HCD conditions or in the presence of synthetic AHLs, expression of the OmpK receptor decreased in *V. anguillarum*, and cell aggregation was reduced [43]. Li et al. (2025) discovered that under conditions of low cell density, QS regulates the expression of BcsE receptor in *Vibrio alginolyticus* [44].

**Fig. 2:**
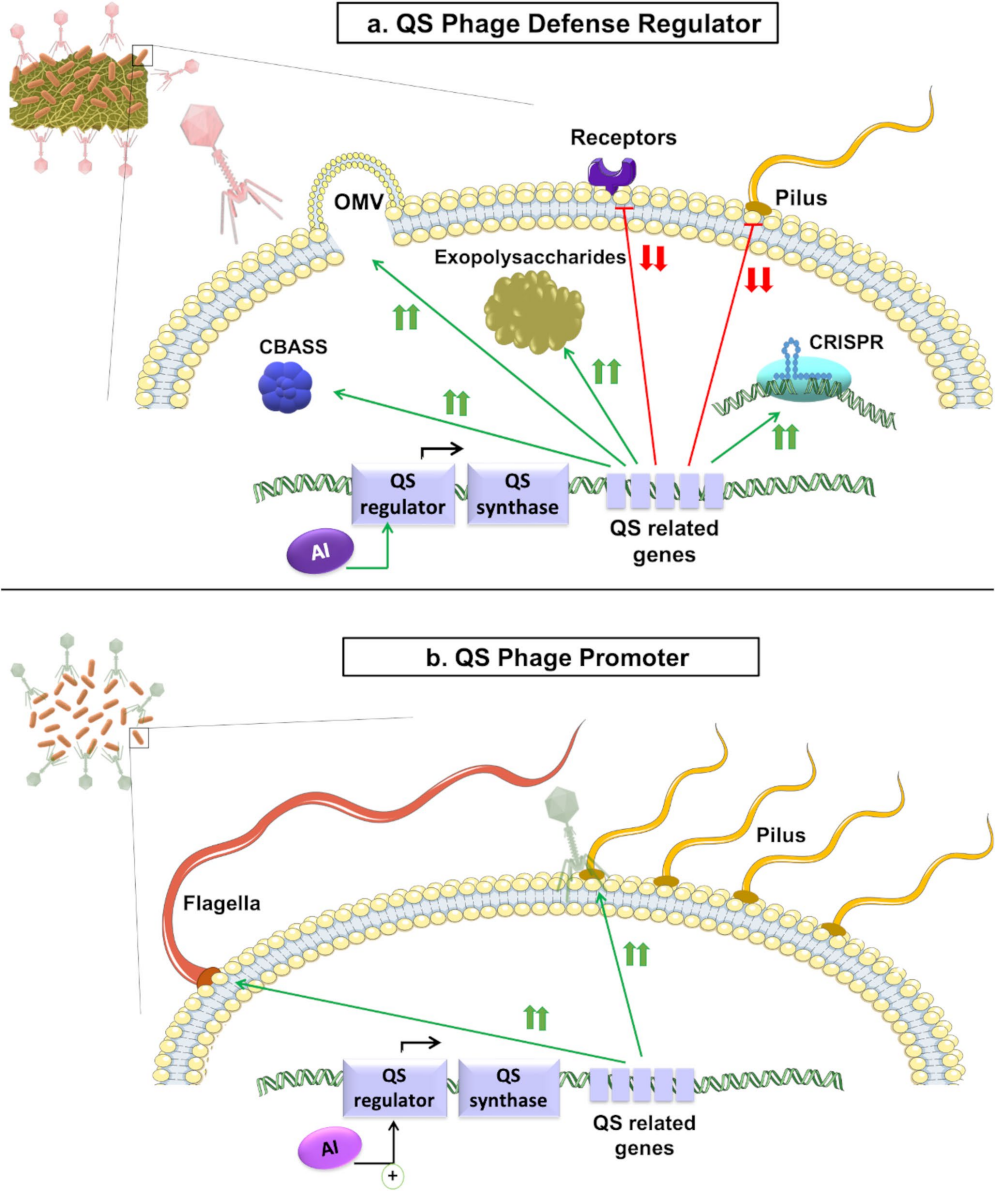
(**a**) QS as a regulator of phage defence. Graphical representation of the mechanism activated by QS to defend bacteria from a phage infection under conditions of high cell density. Green arrows denote activation, while red lines represent inhibition. (**b**)QS as a promoter of phage infection. Graphical representation of the mechanism activated by QS that promotes phage infection during early stages of HCD. Green arrows indicate activation

The bacterial strategies used to hide phage receptor proteins also include the production of outer-membrane vesicles (OMVs) and the formation of capsules and biofilms (Fig. 2a). OMV production is a QS-dependent bacterial decoy that deflects phage attack, as the OMVs are produced when the phage is adsorbed to the bacterial surface, and phages then infect the OMVs instead of the actual bacteria [8, 45]. Biofilm production is strictly dependent on cell density and, therefore, on QS. Biofilms are bacterial communities in which cells are joined to each other and to surfaces by a matrix of extracellular polymeric substances, including polysaccharides, lipids, nucleic acids, and proteins [46, 47]. The polysaccharide matrix protects bacteria against adverse environments and several antimicrobial agents [48]. The increased production of biofilm creates a barrier that reduces phage access to their bacterial receptors [49]. Furthermore, *Enterococcus faecalis* QS controls the production of gelatinase GelE, as a bacterial defence mechanism. The GelE is a secreted metalloprotease, encoded in the Agr-like operon (Fsr cluster), which is directly activated by bacterial AIs, considered essential for induction of biofilm formation in *Enterococcus* sp., as well as being related to virulence [50, 51]. Sheriff et al. (2024) observed significant downexpression of GelE during phage infection, hypothesizing that phages reduce biofilm formation by repressing *gelE*, which produces downregulation of *lrgA* (murein hydrolase regulator) and *lrgB* (antiholin), both involved in protection from extracellular lysis of phages [52]. The previously mentioned SdiA regulator has also been related to biofilm production in the presence of external AHLs, both in *E. coli* and *K. pneumoniae*, reducing phage accessibility [39, 40]. Moreover, phage BUCT640 of *P. aeruginosa* uses the type Psl exopolysaccharides (which are highly dependent on QS) as receptors. The authors demonstrated that the susceptibility of *P. aeruginosa* QS mutants to the phage is significantly increased, showing that QS increases phage resistance by regulating biofilm production through Psl maturation [53].

Bacterial QS has been related to membrane receptors and biofilm production, and also to internal defence systems such as CBASS (Cyclic oligonucleotide-based antiphage signalling system) and the CRISPR-Cas. The CBASS anti-phage defence system is an abortive infection-type system, which is formed by a cyclase, an effector protein, and, in most cases, accessory genes involved in cyclase regulation [54]. Activation of the cyclase produced cycled-nucleotides as second messengers, triggering effector proteins, causing reduction of cell energy, degradation of DNA, or disruption of the bacterial membrane, inhibiting phage propagation [55]. Hoque et al. (2016) observed QS-dependent overexpression of haemagglutinin protease (HAP), which reduced phage susceptibility by significantly decreasing phage stability, presumably by degrading phage particles. Additionally, the HapR regulator has been shown to upregulate the CBASS promoter in *V. cholerae* [54]. The authors demonstrated that under HCD conditions, QS enhances HapR, which activates the CBASS system, increasing anti-phage defence [56].

CRISPR-Cas systems are considered to represent an adaptive immune system in bacteria [57, 58]. These systems consist of repeat arrays separated by small foreign DNA known as spacers. These spacers are acquired by Cas proteins, which cut short fragments of foreign DNA, and are incorporated into the CRISPR array [59, 60]. CRISPR-Cas systems are classified into 6 types, divided into two classes depending on whether they use multi-subunit Cas proteins or a multi-domain Cas as the effector [61]. CRISPR-Cas systems react to previously encountered foreign DNA and cleave it, preventing phage replication [60]. Patterson et al. (2016) reported that under HCD conditions, QS promotes CRISPR-Cas immunity by enhancing spacer acquisition [62]. The ecological significance of QS interaction with CRISPR-Cas was explored by Goldberg et al. (2014), who demonstrated that CRISPR-Cas type III-A plays a role in controlling the induction of lysogenic phages in a transcription-dependent manner, avoiding self-targeting [63]. Although involving plasmids, Høyland-Kroghsbo et al. (2017) demonstrated the link between CRISPR-Cas activity and QS in *P. aeruginosa.* These authors observed a loss of the plasmid both in HCD conditions and when the media was supplemented with AIs, suggesting that CRISPR-Cas regulation by QS balances the cost/benefit ratio of CRISPR-Cas activation during bacterial growth [64]. According to Vale et al. (2005), activation of CRISPR-Cas systems is highly expensive for bacteria, creating a delicate energy balance when phages are involved [65]. Thus, in conditions where there are a large number of phages per bacteria, resistance by elimination of surface receptors is less costly; also, when there are very few phages per bacteria, activation of CRISPR-Cas could lead to autoimmunity [65, 66]. Similar studies have shown QS regulation of CRISPR-Cas activity, and in several of these, no relationship was observed between AI-2 presence and CRISPR-Cas mediated phage-defence in *E. coli* [67]. It has been suggested that QS adaptive immunity is a widespread mechanism across bacterial species under HCD conditions [62].

Bacteria can defend themselves by slowing their metabolism and entering a state of dormancy that prevents phage multiplication. In this respect, it has been shown that in the presence of AI-2, *E. coli* reduces cell metabolism through LsrB and not only downregulating T4P receptor [67]. Additionally, a QS-dependent phage-resistance evolution was observed in *P. aeruginosa*. The authors found that quinolones activate the alkyl-quinolone QS system and thus increase phage resistance. They suggested that external quinolones may be involved in phage resistance evolution either through activation of phage resistance mechanisms or by decreasing the cost of resistance in cells lacking a complete QS and thus altering the balance between cooperation and “cheating” in the population [68].

### QS as a promoter of phage infection

Despite the above findings, QS has also been found to promote phage infection under certain conditions. The increase in phage infection is usually related to the fact that QS promotes the expression of some of the genes used by phages as receptors (Fig. 2b). The effect of QS as a global regulator in bacteria, together with the fact that phages use structural proteins, can cause this contradiction. Therefore, inhibition of QS would lead to a subsequent reduction in the number of phage receptors on the cell surface, thus decreasing the infectivity. Analysis of the behaviour of phage-resistant mutants of *V. alginolyticus* in response to phages Athena1 and VaphiSt2 revealed that several genes related to QS synthesis and detection were downregulated relative to the sensitive wild-type strain, showing that spontaneous mutants inhibit QS as a phage defence mechanism. These researchers found that the two-component sensor systems for AHL and AI-2 were inhibited in both of the mutants analysed, while in one of them (Athena1-resistant), deletion also occurred in the QS two-component CAI-1 histidine kinase sensing system and affected the *cqsS* gene. The researchers also found that many virulence factors were depleted in both of the phage-resistant mutants (Athena 1 and VaphiSt2 resistant-mutants) [69].

In contrast to previous findings in *P. aeruginosa*, it has been observed that inhibition of QS by Baicalein, a flavonoid compound extracted from plants [70], generates a reduction in pilus-dependent phage infection as well as an increase in CRISPR-Cas immunity [71]. Baicalein has been demonstrated to increase lysis of QS-receptor TraR in *E. coli* [72] and reduce expression of QS-genes in *P. aeruginosa* [73]. The authors noted that in the presence of this QS inhibitor, the amount of phage DMS3vir receptors decreased, also reducing the elimination of phages due to CRISPR-Cas activity caused by a reduction in phage adsorption rates. However, they found that in the presence of Baicalein and a high titre of phages, more cells were resistant due to CRISPR immunity than in the control. The authors also found that Baicalein promotes sensitive bacteria, through regulation of T4P-dependent genes when cocultured with receptor-lacking strains [71]. Likewise, *Pseudomonas aeruginosa* responds to environmental indole by reducing the availability of phage receptor T4P (type IV pilus). However, it was not established whether the deficit in the receptor was due to downregulation of the gene expression or inhibition of proper pili assembly [74]. Xuan et al. (2022) also observed an increase in phage adsorption in *P. aeruginosa* due to QS upregulation of GalU, a protein essential for lipopolysaccharide synthesis, and therefore in phage receptors. These researchers demonstrated the effect of lactose inhibitor (*lacI*) in upregulating *galU* and thus boosting phage adsorption [75]. Recently, Cao et al. (2024) found that LasR in *P. aeruginosa* also promotes phage infection by increasing the synthesis of T4P receptor [53]. Moreover, the SdiA receptor can promote phage infection in *Cronobacter sakazakii* by reducing surface hydrophobicity and extracellular matrix, which reduces biofilm formation and increases cell motility, thereby increasing accessibility to the receptors needed by the phage [76]. It has also been shown that in the presence of AI-2, *V. cholerae* activates VqmR, which represses biofilm formation, in turn, increasing access of phages to their host [77].

### Communication by a QS-like system in phages

**Fig. 3:**
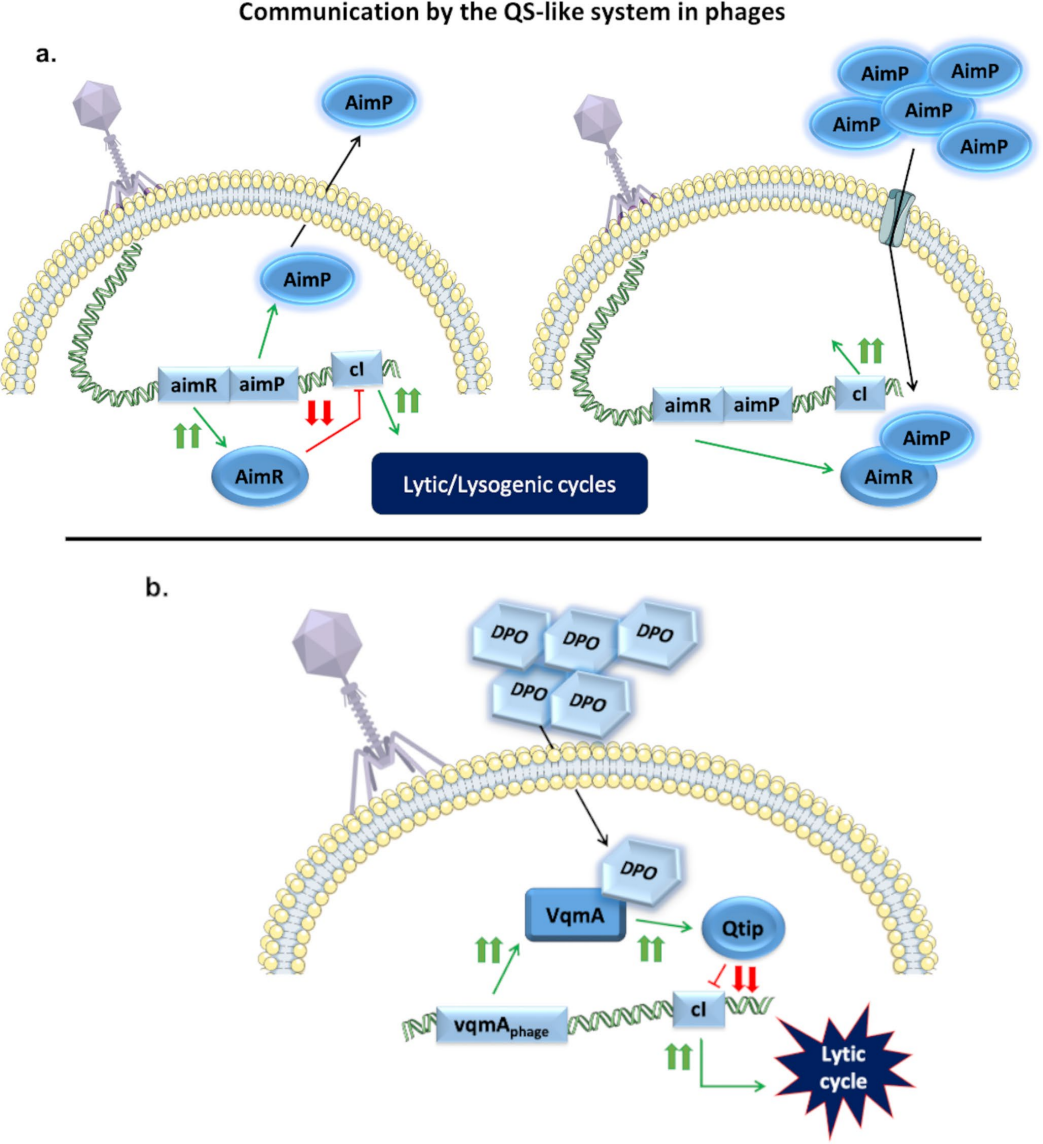
Communication by the QS-like system in phages.(**a**) Graphical representation of the arbitrium system. The image on the left represents the induction of the lytic cycle at low phage density. The image on the right shows the induction of the lysogenic cycle under high phage density conditions. (**b**) VqmA_phage_ system. Representation of how phage codifies VqmA and controls the choice between the lytic and lysogenic cycle on the basis of bacterial cell density. Green arrows indicate activation and synthesis, while red lines denote repression

Improved understanding of the phage-bacteria relationship has led to the discovery of ways in which phages monitor and even use bacterial QS to their advantage. The arbitrium system is known as a phage QS-like communication system (Fig. 3a) [78]. This system, found in 72% of phages in the SPbeta group, allows the phages to enter the lytic or lysogenic cycle depending on the amount of recent infections in the population. Briefly, phages express *aimR* and *aimP* as early genes during infection; AimR activates *aimX*, which is a lysogeny inhibitor, while AimP is processed into a mature signalling peptide that is secreted from the host and incorporated in the rest of the cells in the population by the OPP transporter. In conditions of high infectivity, high levels of AimP will be secreted by the bacteria, thus blocking AimX activity, and the lysogenic cycle will therefore take place [78, 79]. However, the Arbitrum system is not the only way in which phages control their lytic-lysogenic cycles according to QS. Vibriophages are known to harbour a homolog of VqmA DPO-binding QS receptor (VqmAPhage). This receptor detects DPO and produces transcription of the antirepressor Qtip, which inhibits the cIVP882 repressor, activating the lytic cycle of the prophage (Fig. 3b) [80]. Thus, Vibriophages that encode VqmAPhage can regulate their life cycles according to when the probability of infection success is higher. In addition, these phages have also been found to control the host QS by using VqmAPhage to activate VqmR expression and therefore VqmA production [80]. Another well-known example of how phages control bacterial QS is the ability of some *Clostridium difficile* prophages to code for Agr genes. Phages have been described to harbour all these genes, except the Agr receptor (AgrA). Hargreaves et al. (2014) suggested that under suitable conditions, prophages could produce an HCD response without the number of bacteria usually required [81]. Recently, a LuxR-type receptor named Apop was discovered in an *Aeromonas popoffii* phage, although the mechanism of action remains unclear [82].

Prophages are known to harbour genes that can regulate the host metabolism when integrated in the bacterial genome, and also protect the host from being infected by other phages [83]. Shah et al. (2021) investigated the ability of the DMS3 prophage from *P. aeruginosa* to inhibit the host QS. This prophage produces an anti-quorum-sensing protein (Aqs1), which binds the LasR regulator as well as the pili synthesis gene PilB at the early stages of infection, thus inducing superinfection exclusion by reducing expression of the phage receptor. On the other hand, inhibition of LasR by Aqs1 was proposed as a way of avoiding QS-related anti-phage defence mechanisms [39, 62, 84]. Similarly, the PfsE protein in filamentous *P. aeruginosa* prophages was shown to inhibit PQS as well as the pili synthesis gene PilC. PfsE binds PqsA, blocking QS signalling at the same time as binding PilC, reducing phage receptor synthesis. This double function, as in the previous case, allows the phage to protect the lysogen and therefore itself by preventing superinfection [85]. Moreover, the *bci* gene of *P. aeruginosa* filamentous prophages was demonstrated to respond to external AHLs and also regulate the host QS. Ambroa et al. (2020) observed that *bci* not only favours phage infectivity but also regulates expression of the QS regulator genes *lasR*,* rhlR*,* qscR*, and *pqsR*. In addition, the presence of this gene was shown to significantly increase the bacterial virulence, increasing pyocyanin levels, biofilm formation, and therefore reducing motility. The *bci* gene was thus suggested to be an important ally to the maintenance of *P. aeruginosa* in the lungs in cystic fibrosis patients [86]. Another example of phages modulating the host QS has been described in the *P. aeruginosa* lytic phage LUZ19, which codes for a QS targeting protein (Qst), which, in turn, interacts with PqsD, directly disrupting the PQS biosynthesis pathway and causing inhibition of the host QS. Moreover, Qst has been shown to interact with acetyl-CoA and thiamine metabolism, inhibiting cell division and reprogramming the host metabolism to promote phage replication. The authors hypothesized that Qst regulates bacterial energy by reducing the cost of both QS mechanisms, affecting phage-defence systems and acetyl-CoA pathways, to redirect energy towards phage propagation [87, 88]. Furthermore, Leblanc et al. (2009) discovered an acyl hydrolase in an *Iodobacter* phage that degrades AHLs and controls the host QS [89].

Phages can control and monitor bacterial QS to their own benefit to avoid bacterial defence mechanisms, redirect bacterial energy, and communicate with each other. However, prophages can also monitor the host QS to decide when to switch from lysogenic to lytic cycle. This activity has been observed in several *E. coli* phages, such as λ phage, which induce the lytic cycle when the host is present in an environment with a high concentration of AHLs [90]; or more recently, the prophage T1, which transcriptional regulator Pir monitors the levels of AI-2 and cAMP in the host to induce the lytic cycle and upregulate holin production, which is needed for degradation of the cell wall in the last states of the lytic cycle. However, this mechanism is predicted to be widely distributed among phages, as Pir homologues have been found in several phage species [91]. Nevertheless, Mauritzen et al. (2023) demonstrated that some *V. anguillarum* prophages inhibited the phage lytic cycle during HCD conditions [92].

Remarkably, it has been suggested that phages create selective pressure towards functional QS systems in bacteria [93, 94]. Lopez et al. (2018) observed the evolution of an *Acinetobacter baumannii* hospital collection in 10 years, from 2000 to 2010. In the 2010 collection, the acquisition of two temperate phages and a complete QS/QQ system was observed in the majority of the population [93]. In addition, Saucedo-Mora et al. (2017) found several *P. aeruginosa* phages that infect preferably functional QS strains rather than their *ΔlasR*/*ΔrhlR* mutants [95]. On the other hand, Davies et al. (2016) showed that some phages (such as *P. aeruginosa* φ4) prefer to infect QS-deficient bacteria involved in chronic infections, positively selected them in the population [96].

The same QS signal can interact with phage infection by reducing both bacterial metabolism and phage receptors. However, the same signal in a different bacterium may cause the opposite effect by increasing the effectiveness of the infection. These contradictory effects can be explained by the fact that phages use important host elements, which are regulated by QS. These findings must be considered when QS molecules are used with phage therapy, without overlooking the previously mentioned ability of some phages to regulate and monitor bacterial QS to their own benefit.

### Community dynamics

The involvement of QS in phage defence is a complicated subject and involves a delicate equilibrium between QS-mediated gene expression and the activation of antiviral defence mechanisms. The QS is regulated in an HCD-dependent manner, and this system can act by favouring or avoiding phage infection. However, HCD conditions usually occur in bacterial populations composed of different strains and in many environments, of various species. Phage infection in heterogeneous populations and the consequent activation of defence mechanisms stimulate changes in the population, affecting the delicate balance. These heterogeneous populations usually contain QS-defective or “cheating” strains, which either lack QS receptors or QS effectors [97, 98]. Ahator et al. (2022) analysed the dynamics of QS and CRISPR-Cas defective strains in a mixed population of *P. aeruginosa* infected with phages. These authors observed a reduction in phage numbers when bacteria were grown in cocultures rather than monocultures, implying compensation of the different mechanisms that are lacking (QS or CRISPR) in the strains through cooperation between different types of bacteria cooperation between diverse types of bacteria. They also suggested that a mixed population may promote the emergence of resistance in bacteria [99]. These findings were corroborated by Li et al. (2025), who observed a reduction in phage abundance in cocultures of *P. aeruginosa* with and without effective QS. These researchers noted that the application of cell-free supernatant, rich in C4-HSL, to the cultures increased the susceptibility to the phage. The researchers suggested that QS is involved in reducing the metabolic activity of the cells, promoting their entry into a persister state, which would reduce the creation of QS-deficient mutants [100, 101]. They also hypothesized that the phage could recognize and synchronize with these persister cells while waiting for metabolic reactivation to resume propagation [101].

On the other hand, several authors examined how the population changes when multiple bacterial species are present in the population, in an attempt to better understand the changes that occur in the body when phage therapy is applied. To this end, Mumford et al. (2017) analysed the effects of *Pseudomonas* phage PT7 in a mixed population of *P. aeruginosa*/*P. aeruginosa* QS-deficient, *Staphylococcus aureus*, and *Stenotrophomonas maltophilia*. They found that the presence of phages can reduce the total number of bacteria, even though the *P. aeruginosa* QS-mutant was significantly more resistant to the phage than the wild-type. Nevertheless, they also observed an increase in the other pathogens when *P. aeruginosa* is targeted. In addition, the authors observed a reduction in phage-resistant appearance in the mixed communities, suggesting that the high cost of adaptation, with the need for competition between pathogens, could favour the effectiveness of phage therapy [102]. Moreover, the dynamics of a population consisting of *P. aeruginosa*, *S. aureus*,* A. baumannii*, and *Burkholderia cenocepacia* in the presence of *Pseudomonas* DMS3vir phage was analysed. Under these conditions, it was observed that *P. aeruginosa* dominates the population in the absence of phage, but it is substituted by *A. baumannii* in the presence of phage. The authors also noted that although *P. aeruginosa* CRISPR-mutant is fitter in the absence of phages, it is almost eradicated from the population when phage is involved. In addition, these authors proposed that targeted phage treatment of a dominant species in a mixed population helps to maintain the population diversity and that the other species in the community will block reinfection [103].

## Phage therapy and qs, implications and perspectives

This section presents a case report showing the effect of phage infection on bacterial QS and highlighting the most promising molecules proposed for enhancing phage therapy.

### Case report

Blasco et al. (2023) presented a case report of a patient with a multidrug-resistant *P. aeruginosa* infection in a prosthetic vascular graft and who was treated with a three-phage cocktail. After several months of infection and multiple antibiotic treatments, the phage therapy was administered in a regimen of one intravenous injection per day for a week, in combination with ceftazidime-avibactam. After administration of the phage therapy, the strain showed a reduction in resistance to both β-lactamics and quinolone antibiotics (previously resistant). The re-sensitized strain was sequenced and compared with the pre-treatment strain; several mutations in virulence-related (secretion systems, oxidative stress), antibiotic-resistant-related (ion transport, efflux pumps), and phage-resistant genes (surface receptors, QS) were observed. This report shows the evolutionary pathway of bacteria towards phage resistance by increasing biofilm production, and several mutations in surface receptors, toxin-antitoxin systems, metabolic genes, and prophages, directly reducing bacterial virulence and increasing antibiotic sensitivity. Importantly, mutations appeared in the PvdQ gene, an acylase that hydrolyses AHL, inhibiting QS [104]. The mutation in PvdQ increased the activation of QS and, allegedly, its regulated phage-defence mechanisms [105].

### Molecules in use

Due to the involvement of QS in the activation of bacterial defence mechanisms, the use of anti-QS agents as treatment has been deeply studied, focusing on five main strategies: (i) inactivation of QS-receptors, (ii) inhibition, (iii) degradation, (iv) blockage of QS signals, and (v) combination of these agents and antibiotics [106, 107]. In light of the importance of QS also in bacterial phage defence, several molecules have been studied to enhance phage therapy by affecting QS. Some of the most promising ones are mentioned below (Table 1).

**Table 1:** Molecules in use and promising genes for enhancing phage treatment

	Name	Origen	Target	Target species	Observed reaction	References
Mycotoxin	Penicillic acid	*Aspergillus flavus*	LasR/RhlR	*P. aeruginosa*	QS reduction and derived resistance mechanisms	[108, 109]
Lactonase	SsoPox-W163I	*Saccharolobus solfataricus*	AHLs/ CRISPR genes	*P. aeruginosa*	Reduction of CRISPR activity and Pyocyanin	[110–112]
Flavonoid	Cinnamaldehyde (CAD)	*Cinnamomum* spp.	AI-2	*K. pneumoniae*	QS reduction and derived resistance mechanisms	[113–116]
Lactonase gene	AiiA	*Bacillus* spp.	AHLs/PilB	*P. aeruginosa*/ *E. coli*	Biofilm reduction and β-galactosidase and QS	[117]
Anti-QS gene	Aqs1	DMS3 phage	LasR	*P. aeruginosa*	QS reduction and derived resistance mechanisms	[84]
QS-targeting gene	Qst	Luz19 phage	PqsD/ Acetyl-CoA	*P. aeruginosa*	Reduction of PQS synthesis	[87]
Phage gene	Gp70.1	PaP3 phage	RpoS	*P. aeruginosa*	Reduction of cell metabolism and stress response	[118]

One of the compounds that was first found to have promising synergy with phage therapy was penicillic acid. This compound was found to increase the susceptibility of *P. aeruginosa* to phages by targeting LasR and RhlR regulators and thus inhibiting QS [108] and, therefore, also phage-resistant mechanisms regulated by QS. The authors also found that the RNA polymerase of the phage was affected by the presence of penicillic acid [109]. Similarly, the QS-inhibition activity of SsoPox-W163I lactonase (an enzyme that degrades the lactone ring of HSLs) [110] was analysed to determine its potential application in phage therapy. SsoPox-W263I significantly decreases secreted factors, like pyocyanin, in *Pseudomonas* spp., as well as biofilm production in *P. aeruginosa* and *Chromobacterium violaceum*, and violacein in the latter. The presence of SsoPox-W263I also produced downexpression of CRISPR-associated genes in both *P. aeruginosa* and *C. violaceum* [111]. The combination of SsoPox-W263I with phages was found to be a promising strategy, as this enzyme reduces virulence and also the appearance of phage-resistant mutants [112]. Cinnamaldehyde (CAD) is another QS inhibitor with potential in phage therapy as it interacts with AI-2 of different species of bacteria [113–116]. The presence of CAD was shown to increase phage susceptibility in *K. pneumoniae* clinical strains, presumably by avoiding the reduction of membrane proteins and the activation of phage-defence systems [116].

Some authors have used genetically modified phages to inhibit QS. The modified phage carries an enzyme able to disrupt biofilm formation by degrading AHLs (AiiA) [117]. It was demonstrated that the modified phage was able to reduce β-galactosidase activity in the bacterial target. These results open up a new pathway in phage therapy, as AiiA can disrupt the QS of several bacterial species [117]. Moreover, several candidate genes could be engineered in phages, such as the previously mentioned Aqs1 [84] and Qst [87], and also the Gp70.1 from *P. aeruginosa* phage PaP3 [118]. Gp70.1 has been shown to inhibit growth in *P. aeruginosa* and *E. coli* by interacting with the RpoS. The authors demonstrated that the presence of Gp70.1 substantially reduced motility, amino acid and sugar metabolism, stress response, and virulence, highlighting its potential as an antibacterial agent targeting the regulation of sigma factor [118]. However, the identification of new potentially therapeutic genes is complicated since most phage gene functions are unknown, and there is no bioinformatic reference [118].

The combination of anti-QS agents and phages has shown promising in vitro results, becoming an encouraging new path to enhance phage therapy and combat multiresistant bacteria. However, the specific mechanisms by which the above compounds work are still unknown, and diverse efficiencies were observed between species or even strains. Moreover, AiiA and Gp70.1 would require phage engineering, increasing the difficulty of their use. Nevertheless, deeper studies would fill these gaps in our knowledge, taking these methods a step closer to their application.

## Conclusion

In this mini-review, multiple interactions between QS and phage infection are considered. The importance of QS in phage defence is highlighted, without disregarding the paradoxical effects on the expression of some phage receptors, and how phages can control or monitor bacterial QS to their benefit. Finally, several molecules with high synergic potential for use with phage therapy are proposed. In addition, a promising approach would be the use of a triple combination of anti-QS agents, phages, and antibiotics, reducing bacterial resistance. Although the interaction between bacterial QS and phages is complex, modifying bacterial QS as a method of enhancing phage therapy is a promising strategy. However, for effective use of QS in phage therapy, it is important to identify which phage to use, mainly in relation to its bacterial receptors.

## Data Availability

No datasets were generated or analysed during the current study.

## References

[1] Hobbs Z, Abedon ST (2016) Diversity of phage infection types and associated terminology: the problem with “lytic or lysogenic.” FEMS Microbiol Lett. 10.1093/femsle/fnw047 26925588 10.1093/femsle/fnw047

[2] Hampton HG, Watson BNJ, Fineran PC (2020) The arms race between bacteria and their phage foes. Nature 577(7790):327–336. 10.1038/s41586-019-1894-8 31942051 10.1038/s41586-019-1894-8

[3] Dadgostar P (2019) Antimicrobial resistance: implications and costs. Infect Drug Resist 12:3903–3910. 10.2147/IDR.S234610 31908502 10.2147/IDR.S234610 PMC6929930

[4] Skurnik M, Alkalay-Oren S, Boon M, Clokie M, Sicheritz-Pontén T, Dąbrowska K et al (2025) Phage therapy. Nat Rev Methods Primers 5(1):9. 10.1038/s43586-024-00377-5

[5] Blasco L, Bleriot I, Fernandez-Grela P, Pano-Pardo JR, Oteo-Iglesias J, Tomas M (2025) Pharmacokinetics and pharmacodynamics studies of phage therapy. Farm Hosp. 10.1016/j.farma.2025.04.003 40345936 10.1016/j.farma.2025.04.003

[6] Lin DM, Koskella B, Lin HC (2017) Phage therapy: an alternative to antibiotics in the age of multi-drug resistance. World J Gastrointest Pharmacol Ther 8(3):162–173. 10.4292/wjgpt.v8.i3.162 28828194 10.4292/wjgpt.v8.i3.162 PMC5547374

[7] Koskella B, Meaden S (2013) Understanding bacteriophage specificity in natural microbial communities. Viruses 5(3):806–823. 10.3390/v5030806 23478639 10.3390/v5030806 PMC3705297

[8] Manning AJ, Kuehn MJ (2011) Contribution of bacterial outer membrane vesicles to innate bacterial defense. BMC Microbiol 11:258. 10.1186/1471-2180-11-258 22133164 10.1186/1471-2180-11-258 PMC3248377

[9] Buckling A, Rainey PB (2002) Antagonistic coevolution between a bacterium and a bacteriophage. Proc Biol Sci 269(1494):931–936. 10.1098/rspb.2001.1945 12028776 10.1098/rspb.2001.1945 PMC1690980

[10] Abedon ST (2012) Spatial vulnerability: bacterial arrangements, microcolonies, and biofilms as responses to low rather than high phage densities. Viruses 4(5):663–687. 10.3390/v4050663 22754643 10.3390/v4050663 PMC3386622

[11] Duddy OP, Bassler BL (2021) Quorum sensing across bacterial and viral domains. PLoS Pathog 17(1):e1009074. 10.1371/journal.ppat.1009074 33411743 10.1371/journal.ppat.1009074 PMC7790227

[12] Tashiro Y, Kawata K, Taniuchi A, Kakinuma K, May T, Okabe S (2012) RelE-mediated dormancy is enhanced at high cell density in *Escherichia coli*. J Bacteriol 194(5):1169–1176. 10.1128/JB.06628-11 22210768 10.1128/JB.06628-11 PMC3294780

[13] Beulig F, Bafna-Ruhrer J, Jensen PE, Kim SH, Patel A, Kandasamy V et al (2025) Trade-off between resistance and persistence in high cell density cultures. mSystems 10(7):e0032325. 10.1128/msystems.00323-25 40511936 10.1128/msystems.00323-25 PMC12282090

[14] Labrie SJ, Samson JE, Moineau S (2010) Bacteriophage resistance mechanisms. Nat Rev Microbiol 8(5):317–327. 10.1038/nrmicro2315 20348932 10.1038/nrmicro2315

[15] Murtazalieva K, Mu A, Petrovskaya A, Finn RD (2024) The growing repertoire of phage anti-defence systems. Trends Microbiol 32(12):1212–1228. 10.1016/j.tim.2024.05.005 38845267 10.1016/j.tim.2024.05.005

[16] Pena RT, Blasco L, Ambroa A, Gonzalez-Pedrajo B, Fernandez-Garcia L, Lopez M et al (2019) Relationship between quorum sensing and secretion systems. Front Microbiol 10:1100. 10.3389/fmicb.2019.01100 31231316 10.3389/fmicb.2019.01100 PMC6567927

[17] Shang J, Wang K, Zhou Q, Wei Y (2025) The role of quorum sensing in phage lifecycle decision: a switch between lytic and lysogenic pathways. Viruses. 10.3390/v17030317 40143247 10.3390/v17030317 PMC11945551

[18] Chu X, Yang Q (2024) Regulatory mechanisms and physiological impacts of quorum sensing in Gram-negative bacteria. Infect Drug Resist 17:5395–5410. 10.2147/IDR.S485388 39654694 10.2147/IDR.S485388 PMC11626961

[19] Rather MA, Gupta K, Mandal M (2021) Microbial biofilm: formation, architecture, antibiotic resistance, and control strategies. Braz J Microbiol 52(4):1701–1718. 10.1007/s42770-021-00624-x 34558029 10.1007/s42770-021-00624-x PMC8578483

[20] Ng WL, Bassler BL (2009) Bacterial quorum-sensing network architectures. Annu Rev Genet 43:197–222. 10.1146/annurev-genet-102108-134304 19686078 10.1146/annurev-genet-102108-134304 PMC4313539

[21] Gupta R, Schuster M (2013) Negative regulation of bacterial quorum sensing tunes public goods cooperation. ISME J 7(11):2159–2168. 10.1038/ismej.2013.109 23823496 10.1038/ismej.2013.109 PMC3806262

[22] Do H, Makthal N, VanderWal AR, Rettel M, Savitski MM, Peschek N et al (2017) Leaderless secreted peptide signaling molecule alters global gene expression and increases virulence of a human bacterial pathogen. Proc Natl Acad Sci U S A 114(40):E8498–E8507. 10.1073/pnas.1705972114 28923955 10.1073/pnas.1705972114 PMC5635878

[23] Lee J, Jayaraman A, Wood TK (2007) Indole is an inter-species biofilm signal mediated by SdiA. BMC Microbiol 7:42. 10.1186/1471-2180-7-42 17511876 10.1186/1471-2180-7-42 PMC1899176

[24] Garcia-Lara J, Shang LH, Rothfield LI (1996) An extracellular factor regulates expression of sdiA, a transcriptional activator of cell division genes in *Escherichia coli*. J Bacteriol 178(10):2742–2748. 10.1128/jb.178.10.2742-2748.1996 8631660 10.1128/jb.178.10.2742-2748.1996 PMC178007

[25] Papenfort K, Bassler BL (2016) Quorum sensing signal-response systems in Gram-negative bacteria. Nat Rev Microbiol 14(9):576–588. 10.1038/nrmicro.2016.89 27510864 10.1038/nrmicro.2016.89 PMC5056591

[26] Zhang L, Li S, Liu X, Wang Z, Jiang M, Wang R et al (2020) Sensing of autoinducer-2 by functionally distinct receptors in prokaryotes. Nat Commun 11(1):5371. 10.1038/s41467-020-19243-5 33097715 10.1038/s41467-020-19243-5 PMC7584622

[27] Li S, Sun H, Li J, Zhao Y, Wang R, Xu L et al (2022) Autoinducer-2 and bile salts induce c-di-GMP synthesis to repress the T3SS via a T3SS chaperone. Nat Commun 13(1):6684. 10.1038/s41467-022-34607-9 36335118 10.1038/s41467-022-34607-9 PMC9637222

[28] Sifri CD (2008) Quorum sensing: bacteria talk sense. Clin Infect Dis 47(8):1070–1076. 10.1086/592072 18781869 10.1086/592072

[29] Déziel E, Lépine F, Milot S, He J, Mindrinos MN, Tompkins RG et al (2004) Analysis of *Pseudomonas aeruginosa* 4-hydroxy-2-alkylquinolines (HAQs) reveals a role for 4-hydroxy-2-heptylquinoline in cell-to-cell communication. Proc Natl Acad Sci U S A 101(5):1339–1344. 10.1073/pnas.0307694100 14739337 10.1073/pnas.0307694100 PMC337054

[30] Zhou L, Zhang Y, Ge Y, Zhu X, Pan J (2020) Regulatory mechanisms and promising applications of quorum sensing-inhibiting agents in control of bacterial biofilm formation. Front Microbiol 11:589640. 10.3389/fmicb.2020.589640 33178172 10.3389/fmicb.2020.589640 PMC7593269

[31] Fan Q, Zuo J, Wang H, Grenier D, Yi L, Wang Y (2022) Contribution of quorum sensing to virulence and antibiotic resistance in zoonotic bacteria. Biotechnol Adv 59:107965. 10.1016/j.biotechadv.2022.107965 35487393 10.1016/j.biotechadv.2022.107965

[32] Dowah ASA, Clokie MRJ (2018) Review of the nature, diversity and structure of bacteriophage receptor binding proteins that target Gram-positive bacteria. Biophys Rev 10(2):535–542. 10.1007/s12551-017-0382-3 29299830 10.1007/s12551-017-0382-3 PMC5899739

[33] Nouwens AS, Beatson SA, Whitchurch CB, Walsh BJ, Schweizer HP, Mattick JS et al (2003) Proteome analysis of extracellular proteins regulated by the las and rhl quorum sensing systems in *Pseudomonas aeruginosa* PAO1. Microbiology (Reading) 149(5):1311–1322. 10.1099/mic.0.25967-0 12724392 10.1099/mic.0.25967-0

[34] Zhao A, Sun J, Liu Y (2023) Understanding bacterial biofilms: from definition to treatment strategies. Front Cell Infect Microbiol 13:1137947. 10.3389/fcimb.2023.1137947 37091673 10.3389/fcimb.2023.1137947 PMC10117668

[35] Mu H, Han F, Wang Q, Wang Y, Dai X, Zhu M (2023) Recent functional insights into the magic role of (p)ppGpp in growth control. Comput Struct Biotechnol J 21:168–175. 10.1016/j.csbj.2022.11.063 36544478 10.1016/j.csbj.2022.11.063 PMC9747358

[36] van Delden C, Comte R, Bally AM (2001) Stringent response activates quorum sensing and modulates cell density-dependent gene expression in *Pseudomonas aeruginosa*. J Bacteriol 183(18):5376–5384. 10.1128/JB.183.18.5376-5384.2001 11514523 10.1128/JB.183.18.5376-5384.2001 PMC95422

[37] Pacheco T, Gomes AEI, Siqueira NMG, Assoni L, Darrieux M, Venter H et al (2021) SdiA, a quorum-sensing regulator, suppresses fimbriae expression, biofilm formation, and quorum-sensing signaling molecules production in *Klebsiella pneumoniae*. Front Microbiol 12:597735. 10.3389/fmicb.2021.597735 34234747 10.3389/fmicb.2021.597735 PMC8255378

[38] Lee J, Maeda T, Hong SH, Wood TK (2009) Reconfiguring the quorum-sensing regulator SdiA of *Escherichia coli* to control biofilm formation via indole and N-acylhomoserine lactones. Appl Environ Microbiol 75(6):1703–1716. 10.1128/AEM.02081-08 19168658 10.1128/AEM.02081-08 PMC2655446

[39] Hoyland-Kroghsbo NM, Maerkedahl RB, Svenningsen SL (2013) A quorum-sensing-induced bacteriophage defense mechanism. MBio 4(1):e00362-00312. 10.1128/mBio.00362-12 10.1128/mBio.00362-12 PMC3624510 23422409

[40] Silva-Bea S, Maseda P, Otero A, Romero M (2025) Regulatory effects on virulence and phage susceptibility revealed by sdiA mutation in *Klebsiella pneumoniae*. Front Cell Infect Microbiol. 10.3389/fcimb.2025.1562402 40182769 10.3389/fcimb.2025.1562402 PMC11966055

[41] Reeves P (1995) Role of O-antigen variation in the immune response. Trends Microbiol 3(10):381–386. 10.1016/s0966-842x(00)88983-0 8564356 10.1016/s0966-842x(00)88983-0

[42] Hoque MM, Naser IB, Bari SM, Zhu J, Mekalanos JJ, Faruque SM (2016) Quorum regulated resistance of *Vibrio cholerae* against environmental bacteriophages. Sci Rep 6:37956. 10.1038/srep37956 27892495 10.1038/srep37956 PMC5124996

[43] Tan D, Svenningsen SL, Middelboe M (2015) Quorum sensing determines the choice of antiphage defense strategy in *Vibrio anguillarum*. MBio 6(3):e00627. 10.1128/mBio.00627-15 26081633 10.1128/mBio.00627-15 PMC4471561

[44] Li X, Liu X, Ma T, Su H, Sui B, Wang L et al (2025) Understanding phage BX-1 resistance in *Vibrio alginolyticus* AP-1 and the role of quorum-sensing regulation. Microbiol Spectr 13(2):e0243524. 10.1128/spectrum.02435-24 39807883 10.1128/spectrum.02435-24 PMC11792527

[45] Mashburn-Warren L, Howe J, Garidel P, Richter W, Steiniger F, Roessle M et al (2008) Interaction of quorum signals with outer membrane lipids: insights into prokaryotic membrane vesicle formation. Mol Microbiol 69(2):491–502. 10.1111/j.1365-2958.2008.06302.x 18630345 10.1111/j.1365-2958.2008.06302.x PMC2615190

[46] Costerton JW, Lewandowski Z, Caldwell DE, Korber DR, Lappin-Scott HM (1995) Microbial biofilms. Annu Rev Microbiol 49:711–745. 10.1146/annurev.mi.49.100195.003431 8561477 10.1146/annurev.mi.49.100195.003431

[47] Vu B, Chen M, Crawford RJ, Ivanova EP (2009) Bacterial extracellular polysaccharides involved in biofilm formation. Molecules 14(7):2535–2554. 10.3390/molecules14072535 19633622 10.3390/molecules14072535 PMC6254922

[48] Preda VG, Sandulescu O (2019) Communication is the key: biofilms, quorum sensing, formation and prevention. Discoveries (Craiova) 7(3):e100. 10.15190/d.2019.13 32309618 10.15190/d.2019.13 PMC7086079

[49] Sutherland IW, Hughes KA, Skillman LC, Tait K (2004) The interaction of phage and biofilms. FEMS Microbiol Lett 232(1):1–6. 10.1016/S0378-1097(04)00041-2 15061140 10.1016/S0378-1097(04)00041-2

[50] Thomas VC, Hiromasa Y, Harms N, Thurlow L, Tomich J, Hancock LE (2009) A fratricidal mechanism is responsible for eDNA release and contributes to biofilm development of *Enterococcus faecalis*. Mol Microbiol 72(4):1022–1036. 10.1111/j.1365-2958.2009.06703.x 19400795 10.1111/j.1365-2958.2009.06703.x PMC2779696

[51] Engelbert M, Mylonakis E, Ausubel FM, Calderwood SB, Gilmore MS (2004) Contribution of gelatinase, serine protease, and fsr to the pathogenesis of *Enterococcus faecalis* endophthalmitis. Infect Immun 72(6):3628–3633. 10.1128/IAI.72.6.3628-3633.2004 15155673 10.1128/IAI.72.6.3628-3633.2004 PMC415677

[52] Sheriff EK, Salvato F, Andersen SE, Chatterjee A, Kleiner M, Duerkop BA (2024) *Enterococcal* quorum-controlled protease alters phage infection. FEMS Microbes 5:xtae022. 10.1093/femsmc/xtae022 39156124 10.1093/femsmc/xtae022 PMC11328733

[53] Cao L, Mi J, He Y, Xuan G, Wang J, Li M et al (2025) Quorum sensing inhibits phage infection by regulating biofilm formation of *P. aeruginosa* PAO1. J Virol 99(2):e0187224. 10.1128/jvi.01872-24 39745428 10.1128/jvi.01872-24 PMC11853092

[54] Millman A, Melamed S, Amitai G, Sorek R (2020) Diversity and classification of cyclic-oligonucleotide-based anti-phage signalling systems. Nat Microbiol 5(12):1608–1615. 10.1038/s41564-020-0777-y 32839535 10.1038/s41564-020-0777-y PMC7610970

[55] Wang L, Zhang L (2023) The arms race between bacteria CBASS and bacteriophages. Front Immunol 14:1224341. 10.3389/fimmu.2023.1224341 37575224 10.3389/fimmu.2023.1224341 PMC10419184

[56] Severin GB, Ramliden MS, Ford KC, Van Alst AJ, Sanath-Kumar R, Decker KA et al (2023) Activation of a *Vibrio cholerae* CBASS anti-phage system by quorum sensing and folate depletion. MBio 14(5):e0087523. 10.1128/mbio.00875-23 37623317 10.1128/mbio.00875-23 PMC10653837

[57] Deveau H, Garneau JE, Moineau S (2010) CRISPR/Cas system and its role in phage-bacteria interactions. Annu Rev Microbiol 64:475–493. 10.1146/annurev.micro.112408.134123 20528693 10.1146/annurev.micro.112408.134123

[58] Mojica FJ, Diez-Villasenor C, Garcia-Martinez J, Soria E (2005) Intervening sequences of regularly spaced prokaryotic repeats derive from foreign genetic elements. J Mol Evol 60(2):174–182. 10.1007/s00239-004-0046-3 15791728 10.1007/s00239-004-0046-3

[59] Makarova KS, Haft DH, Barrangou R, Brouns SJ, Charpentier E, Horvath P et al (2011) Evolution and classification of the CRISPR-Cas systems. Nat Rev Microbiol 9(6):467–477. 10.1038/nrmicro2577 21552286 10.1038/nrmicro2577 PMC3380444

[60] Barrangou R, Fremaux C, Deveau H, Richards M, Boyaval P, Moineau S et al (2007) CRISPR provides acquired resistance against viruses in prokaryotes. Science 315(5819):1709–1712. 10.1126/science.1138140 17379808 10.1126/science.1138140

[61] de González Aledo M, González-Bardanca M, Blasco L, Pacios O, Bleriot I, Fernández-García L et al (2021) CRISPR-Cas, a revolution in the treatment and study of ESKAPE infections: pre-clinical studies. Antibiotics. 10.3390/antibiotics10070756 10.3390/antibiotics10070756 PMC8300728 34206474

[62] Patterson AG, Jackson SA, Taylor C, Evans GB, Salmond GPC, Przybilski R et al (2016) Quorum sensing controls adaptive immunity through the regulation of multiple CRISPR-Cas systems. Mol Cell 64(6):1102–1108. 10.1016/j.molcel.2016.11.012 27867010 10.1016/j.molcel.2016.11.012 PMC5179492

[63] Goldberg GW, Jiang W, Bikard D, Marraffini LA (2014) Conditional tolerance of temperate phages via transcription-dependent CRISPR-Cas targeting. Nature 514(7524):633–637. 10.1038/nature13637 25174707 10.1038/nature13637 PMC4214910

[64] Hoyland-Kroghsbo NM, Paczkowski J, Mukherjee S, Broniewski J, Westra E, Bondy-Denomy J et al (2017) Quorum sensing controls the *Pseudomonas aeruginosa* CRISPR-Cas adaptive immune system. Proc Natl Acad Sci U S A 114(1):131–135. 10.1073/pnas.1617415113 27849583 10.1073/pnas.1617415113 PMC5224376

[65] Vale PF, Lafforgue G, Gatchitch F, Gardan R, Moineau S, Gandon S (2015) Costs of CRISPR-Cas-mediated resistance in *Streptococcus thermophilus*. Proc Biol Sci 282(1812):20151270. 10.1098/rspb.2015.1270 26224708 10.1098/rspb.2015.1270 PMC4528535

[66] Stern A, Keren L, Wurtzel O, Amitai G, Sorek R (2010) Self-targeting by CRISPR: gene regulation or autoimmunity? Trends Genet 26(8):335–340. 10.1016/j.tig.2010.05.008 20598393 10.1016/j.tig.2010.05.008 PMC2910793

[67] Ding Y, Zhang D, Zhao X, Tan W, Zheng X, Zhang Q et al (2021) Autoinducer-2-mediated quorum-sensing system resists T4 phage infection in *Escherichia coli*. J Basic Microbiol 61(12):1113–1123. 10.1002/jobm.202100344 34783039 10.1002/jobm.202100344

[68] Moreau P, Diggle SP, Friman VP (2017) Bacterial cell-to-cell signaling promotes the evolution of resistance to parasitic bacteriophages. Ecol Evol 7(6):1936–1941. 10.1002/ece3.2818 28331600 10.1002/ece3.2818 PMC5355186

[69] Skliros D, Droubogiannis S, Kalloniati C, Katharios P, Flemetakis E (2023) Perturbation of quorum sensing after the acquisition of bacteriophage resistance could contribute to novel traits in *Vibrio alginolyticus*. Microorganisms. 10.3390/microorganisms11092273 37764117 10.3390/microorganisms11092273 PMC10535087

[70] Sabry K, Jamshidi Z, Emami SA, Sahebka A (2024) Potential therapeutic effects of baicalin and baicalein. Avicenna J Phytomed 14(1):23–49. 10.22038/AJP.2023.22307 38948180 10.22038/AJP.2023.22307 PMC11210699

[71] Broniewski JM, Chisnall MAW, Hoyland-Kroghsbo NM, Buckling A, Westra ER (2021) The effect of quorum sensing inhibitors on the evolution of CRISPR-based phage immunity in *Pseudomonas aeruginosa*. ISME J 15(8):2465–2473. 10.1038/s41396-021-00946-6 33692485 10.1038/s41396-021-00946-6 PMC8319334

[72] Zeng Z, Qian L, Cao L, Tan H, Huang Y, Xue X et al (2008) Virtual screening for novel quorum sensing inhibitors to eradicate biofilm formation of *Pseudomonas aeruginosa*. Appl Microbiol Biotechnol 79(1):119–126. 10.1007/s00253-008-1406-5 18330563 10.1007/s00253-008-1406-5

[73] Luo J, Kong JL, Dong BY, Huang H, Wang K, Wu LH et al (2016) Baicalein attenuates the quorum sensing-controlled virulence factors of *Pseudomonas aeruginosa* and relieves the inflammatory response in *P. aeruginosa*-infected macrophages by downregulating the MAPK and NFkappaB signal-transduction pathways. Drug Des Devel Ther 10:183–203. 10.2147/DDDT.S97221 26792984 10.2147/DDDT.S97221 PMC4708194

[74] Xuan G, Dou Q, Kong J, Lin H, Wang J (2023) *Pseudomonas aeruginosa* resists phage infection via eavesdropping on Indole signaling. Microbiol Spectr 11(1):e0391122. 10.1128/spectrum.03911-22 36602321 10.1128/spectrum.03911-22 PMC9927445

[75] Xuan G, Lin H, Tan L, Zhao G, Wang J (2022) Quorum sensing promotes phage infection in *Pseudomonas aeruginosa*PAO1. MBio 13(1):e0317421. 10.1128/mbio.03174-21 35038901 10.1128/mbio.03174-21 PMC8764535

[76] Cao Y, Li L, Zhang Y, Liu F, Xiao X, Li X et al (2022) Evaluation of *Cronobacter sakazakii* biofilm formation after sdiA knockout in different osmotic pressure conditions. Food Res Int 151:110886. 10.1016/j.foodres.2021.110886 34980413 10.1016/j.foodres.2021.110886

[77] Papenfort K, Forstner KU, Cong JP, Sharma CM, Bassler BL (2015) Differential RNA-seq of *Vibrio cholerae* identifies the VqmR small RNA as a regulator of biofilm formation. Proc Natl Acad Sci U S A 112(7):E766-775. 10.1073/pnas.1500203112 25646441 10.1073/pnas.1500203112 PMC4343088

[78] Erez Z, Steinberger-Levy I, Shamir M, Doron S, Stokar-Avihail A, Peleg Y et al (2017) Communication between viruses guides lysis-lysogeny decisions. Nature 541(7638):488–493. 10.1038/nature21049 28099413 10.1038/nature21049 PMC5378303

[79] Stokar-Avihail A, Tal N, Erez Z, Lopatina A, Sorek R (2019) Widespread utilization of peptide communication in phages infecting soil and pathogenic bacteria. Cell Host Microbe 25(5):746–755. 10.1016/j.chom.2019.03.017 31071296 10.1016/j.chom.2019.03.017 PMC6986904

[80] Silpe JE, Bassler BL (2019) A host-produced quorum-sensing autoinducer controls a phage lysis-lysogeny decision. Cell 176(1–2):268–280. 10.1016/j.cell.2018.10.059 30554875 10.1016/j.cell.2018.10.059 PMC6329655

[81] Hargreaves KR, Kropinski AM, Clokie MR (2014) What does the talking?: quorum sensing signalling genes discovered in a bacteriophage genome. PLoS One 9(1):e85131. 10.1371/journal.pone.0085131 24475037 10.1371/journal.pone.0085131 PMC3901668

[82] Silpe JE, Bassler BL (2019) Phage-encoded LuxR-type receptors responsive to host-produced bacterial quorum-sensing autoinducers. MBio. 10.1128/mBio.00638-19 30967469 10.1128/mBio.00638-19 PMC6456758

[83] Fortier LC, Sekulovic O (2013) Importance of prophages to evolution and virulence of bacterial pathogens. Virulence 4(5):354–365. 10.4161/viru.24498 23611873 10.4161/viru.24498 PMC3714127

[84] Shah M, Taylor VL, Bona D, Tsao Y, Stanley SY, Pimentel-Elardo SM et al (2021) A phage-encoded anti-activator inhibits quorum sensing in *Pseudomonas aeruginosa*. Mol Cell 81(3):571–583e576. 10.1016/j.molcel.2020.12.011 33412111 10.1016/j.molcel.2020.12.011

[85] Schwartzkopf CM, Taylor VL, Groleau MC, Faith DR, Schmidt AK, Lamma TL et al (2024) Inhibition of PQS signaling by the Pf bacteriophage protein PfsE enhances viral replication in *Pseudomonas aeruginosa*. Mol Microbiol 121(1):116–128. 10.1111/mmi.15202 38038061 10.1111/mmi.15202 PMC10842821

[86] Ambroa A, Blasco L, Lopez-Causape C, Trastoy R, Fernandez-Garcia L, Bleriot I et al (2020) Temperate bacteriophages (prophages) in *Pseudomonas aeruginosa* isolates belonging to the International Cystic Fibrosis Clone (CC274). Front Microbiol 11:556706. 10.3389/fmicb.2020.556706 33101229 10.3389/fmicb.2020.556706 PMC7546807

[87] Hendrix H, Zimmermann-Kogadeeva M, Zimmermann M, Sauer U, De Smet J, Muchez L et al (2022) Metabolic reprogramming of *Pseudomonas aeruginosa* by phage-based quorum sensing modulation. Cell Rep 38(7):110372. 10.1016/j.celrep.2022.110372 35172131 10.1016/j.celrep.2022.110372

[88] Schmidt AK, Fitzpatrick AD, Schwartzkopf CM, Faith DR, Jennings LK, Coluccio A et al (2022) A filamentous bacteriophage protein inhibits type IV pili to prevent superinfection of *Pseudomonas aeruginosa*. MBio 13(1):e0244121. 10.1128/mbio.02441-21 35038902 10.1128/mbio.02441-21 PMC8764522

[89] Leblanc C, Caumont-Sarcos A, Comeau AM, Krisch HM (2009) Isolation and genomic characterization of the first phage infecting *Iodobacteria*: varphiPLPE, a myovirus having a novel set of features. Environ Microbiol Rep 1(6):499–509. 10.1111/j.1758-2229.2009.00055.x 23765928 10.1111/j.1758-2229.2009.00055.x

[90] Ghosh D, Roy K, Williamson KE, Srinivasiah S, Wommack KE, Radosevich M (2009) Acyl-homoserine lactones can induce virus production in lysogenic bacteria: an alternative paradigm for prophage induction. Appl Environ Microbiol 75(22):7142–7152. 10.1128/AEM.00950-09 19783745 10.1128/AEM.00950-09 PMC2786502

[91] Laganenka L, Sander T, Lagonenko A, Chen Y, Link H, Sourjik V (2019) Quorum sensing and metabolic state of the host control lysogeny-lysis switch of bacteriophage T1. MBio. 10.1128/mBio.01884-19 31506310 10.1128/mBio.01884-19 PMC6737242

[92] Mauritzen JJ, Sondberg E, Kalatzis PG, Roager L, Gram L, Svenningsen SL et al (2023) Strain-specific quorum-sensing responses determine virulence properties in *Vibrio anguillarum*. Environ Microbiol 25(7):1344–1362. 10.1111/1462-2920.16356 36807464 10.1111/1462-2920.16356

[93] López M, Rueda A, Florido JP, Blasco L, Fernández-García L, Trastoy R et al (2018) Evolution of the quorum network and the mobilome (plasmids and bacteriophages) in clinical strains of *Acinetobacter baumannii* during a decade. Sci Rep 8(1):2523. 10.1038/s41598-018-20847-7 29410443 10.1038/s41598-018-20847-7 PMC5802823

[94] Leon-Felix J, Villicana C (2021) The impact of quorum sensing on the modulation of phage-host interactions. J Bacteriol 203(9). 10.1128/JB.00687-20 10.1128/JB.00687-20 PMC8117525 33468597

[95] Saucedo-Mora MA, Castaneda-Tamez P, Cazares A, Perez-Velazquez J, Hense BA, Cazares D et al (2017) Selection of functional quorum sensing systems by lysogenic bacteriophages in *Pseudomonas aeruginosa*. Front Microbiol 8:1669. 10.3389/fmicb.2017.01669 28912771 10.3389/fmicb.2017.01669 PMC5583629

[96] Davies EV, James CE, Williams D, O’Brien S, Fothergill JL, Haldenby S et al (2016) Temperate phages both mediate and drive adaptive evolution in pathogen biofilms. Proc Natl Acad Sci U S A 113(29):8266–8271. 10.1073/pnas.1520056113 27382184 10.1073/pnas.1520056113 PMC4961188

[97] Diggle SP, Griffin AS, Campbell GS, West SA (2007) Cooperation and conflict in quorum-sensing bacterial populations. Nature 450(7168):411–414. 10.1038/nature06279 18004383 10.1038/nature06279

[98] Asfahl KL, Schuster M (2017) Social interactions in bacterial cell-cell signaling. FEMS Microbiol Rev 41(1):92–107. 10.1093/femsre/fuw038 27677972 10.1093/femsre/fuw038

[99] Ahator SD, Sagar S, Zhu M, Wang J, Zhang LH (2022) Nutrient availability and phage exposure alter the quorum-sensing and CRISPR-Cas-controlled population dynamics of *Pseudomonas aeruginosa*. mSystems 7(4):e0009222. 10.1128/msystems.00092-22 35699339 10.1128/msystems.00092-22 PMC9426516

[100] Dandekar AA, Chugani S, Greenberg EP (2012) Bacterial quorum sensing and metabolic incentives to cooperate. Science 338(6104):264–266. 10.1126/science.1227289 23066081 10.1126/science.1227289 PMC3587168

[101] Li D, Li N, Chen Y, Yang Y, Pan J, Lin J et al (2025) Phage-host interaction in *Pseudomonas aeruginosa* clinical isolates with functional and altered quorum sensing systems. Appl Environ Microbiol 91(4):e0240224. 10.1128/aem.02402-24 40035599 10.1128/aem.02402-24 PMC12016573

[102] Mumford R, Friman VP (2017) Bacterial competition and quorum-sensing signalling shape the eco-evolutionary outcomes of model in vitro phage therapy. Evol Appl 10(2):161–169. 10.1111/eva.12435 28127392 10.1111/eva.12435 PMC5253424

[103] Alseth EO, Custodio R, Sundius SA, Kuske RA, Brown SP, Westra ER (2024) The impact of phage and phage resistance on microbial community dynamics. PLoS Biol 22(4):e3002346. 10.1371/journal.pbio.3002346 38648198 10.1371/journal.pbio.3002346 PMC11034675

[104] Sio CF, Otten LG, Cool RH, Diggle SP, Braun PG, Bos R et al (2006) Quorum quenching by an N-acyl-homoserine lactone acylase from *Pseudomonas aeruginosa* PAO1. Infect Immun 74(3):1673–1682. 10.1128/IAI.74.3.1673-1682.2006 16495538 10.1128/IAI.74.3.1673-1682.2006 PMC1418629

[105] Blasco L, López-Hernández I, Rodríguez-Fernández M, Pérez-Florido J, Casimiro-Soriguer CS, Djebara S et al (2023) Case report: analysis of phage therapy failure in a patient with a *Pseudomonas aeruginosa* prosthetic vascular graft infection. Front Med (Lausanne) 10:1199657. 10.3389/fmed.2023.1199657 37275366 10.3389/fmed.2023.1199657 PMC10235614

[106] Jiang Q, Chen J, Yang C, Yin Y, Yao K (2019) Quorum sensing: a prospective therapeutic target for bacterial diseases. BioMed Res Int 2019:2015978. 10.1155/2019/2015978 31080810 10.1155/2019/2015978 PMC6475571

[107] Zhao X, Yu Z, Ding T (2020) Quorum-sensing regulation of antimicrobial resistance in bacteria. Microorganisms. 10.3390/microorganisms8030425 32192182 10.3390/microorganisms8030425 PMC7143945

[108] Rasmussen TB, Skindersoe ME, Bjarnsholt T, Phipps RK, Christensen KB, Jensen PO et al (2005) Identity and effects of quorum-sensing inhibitors produced by *Penicillium* species. Microbiology (Reading) 151(Pt 5):1325–1340. 10.1099/mic.0.27715-0 15870443 10.1099/mic.0.27715-0

[109] Qin X, Sun Q, Yang B, Pan X, He Y, Yang H (2017) Quorum sensing influences phage infection efficiency via affecting cell population and physiological state. J Basic Microbiol 57(2):162–170. 10.1002/jobm.201600510 27714824 10.1002/jobm.201600510

[110] Hiblot J, Gotthard G, Elias M, Chabriere E (2013) Differential active site loop conformations mediate promiscuous activities in the lactonase SsoPox. PLoS One 8(9):e75272. 10.1371/journal.pone.0075272 24086491 10.1371/journal.pone.0075272 PMC3781021

[111] Mion S, Plener L, Remy B, Daude D, Chabriere E (2019) Lactonase SsoPox modulates CRISPR-Cas expression in gram-negative Proteobacteria using AHL-based quorum sensing systems. Res Microbiol 170(6–7):296–299. 10.1016/j.resmic.2019.06.004 31279087 10.1016/j.resmic.2019.06.004

[112] Mion S, Remy B, Plener L, Bregeon F, Chabriere E, Daude D (2019) Quorum quenching lactonase strengthens bacteriophage and antibiotic arsenal against *Pseudomonas aeruginosa*Clinical isolates. Front Microbiol 10:2049. 10.3389/fmicb.2019.02049 31551983 10.3389/fmicb.2019.02049 PMC6734170

[113] Brackman G, Hillaert U, Van Calenbergh S, Nelis HJ, Coenye T (2009) Use of quorum sensing inhibitors to interfere with biofilm formation and development in *Burkholderia multivorans* and *Burkholderia cenocepacia*. Res Microbiol 160(2):144–151. 10.1016/j.resmic.2008.12.003 19146953 10.1016/j.resmic.2008.12.003

[114] Brackman G, Celen S, Hillaert U, Van Calenbergh S, Cos P, Maes L et al (2011) Structure-activity relationship of cinnamaldehyde analogs as inhibitors of AI-2 based quorum sensing and their effect on virulence of *Vibrio* spp. PLoS One 6(1):e16084. 10.1371/journal.pone.0016084 21249192 10.1371/journal.pone.0016084 PMC3020944

[115] Capper-Parkin KL, Nichol T, Smith TJ, Lacey MM, Forbes S (2023) Antimicrobial and cytotoxic synergism of biocides and quorum-sensing inhibitors against uropathogenic *Escherichia coli*. J Hosp Infect 134:138–146. 10.1016/j.jhin.2023.02.004 36801429 10.1016/j.jhin.2023.02.004

[116] Barrio-Pujante A, Bleriot I, Blasco L, Fernández-Garcia L, Pacios O, Ortiz-Cartagena C et al (2024) Regulation of anti-phage defense mechanisms by using cinnamaldehyde as a quorum sensing inhibitor. Front Microbiol 15:1416628. 10.3389/fmicb.2024.1416628 38989015 10.3389/fmicb.2024.1416628 PMC11233531

[117] Pei R, Lamas-Samanamud GR (2014) Inhibition of biofilm formation by T7 bacteriophages producing quorum-quenching enzymes. Appl Environ Microbiol 80(17):5340–5348. 10.1128/AEM.01434-14 24951790 10.1128/AEM.01434-14 PMC4136088

[118] Zhao X, Chen C, Jiang X, Shen W, Huang G, Le S et al (2016) Transcriptomic and metabolomic analysis revealed multifaceted effects of phage protein Gp70.1 on *Pseudomonas aeruginosa*. Front Microbiol 7:1519. 10.3389/fmicb.2016.01519 27725812 10.3389/fmicb.2016.01519 PMC5035744

